# Unveiling the Effects of Stressors on Task Performance: The Role of Thriving at Work and Resilience

**DOI:** 10.3389/fpsyg.2022.896505

**Published:** 2022-05-30

**Authors:** Zahid Hussain, Hasan Farid, Xinran Liu, Wasim Abbass Shaheen

**Affiliations:** ^1^School of Finance, Qilu University of Technology (Shandong Academy of Sciences), Jinan, China; ^2^Business School, Hohai University, Nanjing, China; ^3^Quaid-i-Azam School of Management Sciences, Quaid-i-Azam University, Islamabad, Pakistan

**Keywords:** role conflict, perceived workload, thriving at work, task performance, resilience, conservation of resources

## Abstract

This study unveils the effects of stressors on employees' task performance through the mediating role of thriving at work (TAW) and a moderating role of resilience (RES) grounding on conservation of resources (COR) theory. The analysis of collected data from 331 supervisor-employee dyads in the hospitality sector of China explicates that the role conflict (RC) and perceived workload have a negative influence on TAW, and thriving has a positive relationship with task performance. The results corroborate the mediating role of TAW between RC, perceived workload, and task performance. Furthermore, the RES suppressed the negative relationship between RC, perceived workload, and TAW. Moreover, our study underscores the theoretical and practical contributions regarding the negative influence of stressors on TAW by exhibiting the importance of the COR mechanism for employees' behavioral outcomes.

## Introduction

Organizations are operating in a competitive era that urges them to be competitive in the global environment (Guo and Chen, 2021). Frontline employees in this regard play a crucial role in gaining a competitive advantage (Kim et al., 2021; Pedersen, 2021). Employees are characterized by job stressors that hamper their learning and vitality (Yang and Li, 2021). Recent studies have urged the need to nourish individuals' psychological states, such as thriving at work (TAW) (Kleine et al., 2019; Farid et al., 2021). Previous studies were centered on positive psychology which ignored the importance of stressors that hinder employees' performance and affect their psychological states negatively (Flinchbaugh et al., 2015). To bridge this gap, we aimed to explore the detrimental effects of role conflict (RC) and perceived workload which can affect employees' thriving negatively, and with a low level of thriving, employees' are not able to exhibit their task performance (Marchiondo et al., 2018).

Role conflict is the contradiction between authorities, messages, and responsibilities (Kawiana et al., 2018), while work overload is referred to performing such tasks which require extra hours to be accomplished (Allen et al., 2008). RC and perceived workload have been evidenced for creating adverse effects on employees' behavioral and attitudinal outcomes, such as work engagement, job satisfaction, organizational commitment, motivation, and performance (Maden-Eyiusta, 2019; Saputra and Surati, 2019; Siswanto et al., 2019; Belias et al., 2021). Employees loaded with stressors may not experience thriving which is the charismatic psychological state for employees to perform better. TAW is a psychological state entailing a joint experience of vitality and learning (Spreitzer et al., 2005). Previous studies have shown a negative association between stressors and TAW (Marchiondo et al., 2018; Nawaz et al., 2018; Yang and Li, 2021; Zhu et al., 2021). Walumbwa et al. (2018) explicated that thriving makes individuals effectively perform their tasks.

This study employs the conservation of resources (COR) theory (Hobfoll, 1989) as the underlying mechanism to explain the relationship among variables of interest. The core tenant of COR theory elucidates the human behavior that is based on the innate desire to gain and conserve the resources for existence (Hobfoll, 2002). COR delineates that resources glean enhanced behavioral outcomes, and stressors (RC and work overload) cause loss in a spiral of resources (Hobfoll et al., 2018). This study fills the gap in the existing body of knowledge in several ways. We investigated the role of stressors [RC and perceived workload (PWL)] and the Chinese hospitality context that was not considered previously among studies on thriving. It also unveils the link between thriving and task performance along with the crucial mediating role of thriving between RC and perceived workload and task performance. This study also examines resilience (RES) as a boundary condition between stressors and thriving work to explain the level of individuals' ability to cope with the negative effects caused by stressors.

## Theory and Hypotheses

### RC, PWL, and TAW

Role conflict is defined as when there is a contradiction between authorities, messages, and responsibilities (Kawiana et al., 2018). An extant body of knowledge realized RC as a job stressor due to its negative influence on psychological and behavioral aspects of individuals (Schmidt et al., 2014). COR theory of Hobfoll (1989) argues that resources play a key role in coping with stress and strengthen the ability to enthusiastically perform work tasks.

Role conflict results in work-related strain, which not only hampers intangible perspectives (Van Dyne et al., 2002) but also causes loss in a spiral of resources (Hobfoll et al., 2018). When individuals are exposed to stressors, they seek coping strategies (Decker and Borgen, 1993). This process of searching for coping strategies restricts them to feel vital (Latack and Havlovic, 1992). Moreover, the stressors impede the process of acquiring knowledge and skills (LePine et al., 2004), which acts as an obstacle to employees' learning (Kleine et al., 2019).

Palomino and Frezatti (2016) stated that RC causes the loss of resources which hampers the accomplishment of job tasks. It also decreases the satisfaction with one's job due to a higher level of anxiety (Tarrant and Sabo, 2010). When individuals face conflict in their job roles, they will not be able to retain resources and also lose the existing pool of resources. Spreitzer et al. (2005) suggested that individuals may not experience a sense of thriving without resource availability which works as an engine of thriving. Past studies have shown that stressful events cause the loss of resources which restricts the individuals' ability to feel vital and learn at work (Flinchbaugh et al., 2015; Cullen et al., 2018).

Work overload is referred to performing such tasks that require extra hours to be accomplished. This situation creates a burden on employees, and they are unable to perform the basic tasks (Allen et al., 2008). Ilies et al. (2007) commented that work overload creates disturbance not only in individuals' behavior at work but also in family times. Kim et al. (2010) stated that employees with heavy workloads feel emotionally burdened that causes psychological distress. Schaufeli et al. (2008) suggested that work overload is a root cause of distress in employees, which affects their working ability (Nguyen et al., 2018).

Work overload bears negative consequences which can cause the loss of individuals' resources. A strong association of work overload with burnout has been evidenced among healthcare employees (Deodhar and Goswami, 2017). A heavy workload as a stressor can make individuals physically and emotionally exhausted. Previous research states that stressors cause loss of resources, which hampers individuals' thriving (Porath et al., 2012; Marchiondo et al., 2018). Hobfoll (2002) commented that individuals with fewer resources face a loss of resources termed as “loss cycle.” This loss cycle means that job stressors make individuals lose their resources, and they do not feel thriving. Excessive time demands and work overloads can lead to declined intrinsic motivation (Chen et al., 2015), which is central to enabling thriving.

Spreitzer et al. (2005) stated that resources play an important part in enabling individuals' thriving. While different types of stressors entail role stressors, hindrance stressors had a negative adverse relationship with thriving (Cullen et al., 2018; Rehmat et al., 2021). Shirom et al. (2006) observed that individuals are not able to attain desired performance while facing stress due to different reasons. Spreitzer et al. (2005) delineated that negative affective resources hamper the individuals' way toward thriving. Employees experiencing conflict in roles and heavy workload may not perceive fairness, due to which they may not thrive at work (Abid et al., 2015).

**H1:** *Role conflict has a negative influence on TAW*.

**H2:** *Perceived workload has a negative influence on TAW*.

### TAW and Task Performance

Thriving employees are in a better position where they utilize their energy and acquire knowledge to perform their assigned tasks efficiently (Frazier and Tupper, 2018). Task performance is an in-role behavior, defined as performing the formal job requirements by following the set organizational procedures. Moreover, it is a nondiscretionary role that has to be performed efficiently and effectively since most of the tasks are included in the routine work of employees (Yu and Frenkel, 2013). In line with the phenomena explained by Griffin et al. (2007) about performance efficiency, Porath et al. (2012) conceptualized performance as an effective way of meeting the organizational expectation of employees at work.

Conservation of resources as a motivational theory state that acquisition and COR are key factors that explain human behavior (Hobfoll et al., 2018). Since thriving individuals possess enough resources which make them fulfill their task performance in a better way, employees using thriving as a gauge for self-development tend to behave agentic and in this course control their destiny in a responsible way which turns into an enhanced performance mechanism (Grant et al., 2011). Individuals acting genetically gain more resources which strengthens their behavior (Hobfoll, 2012). The tendency of thriving employees in gaining more resources creates more knowledge and strong relationships and develops meaning while executing job tasks (Spreitzer et al., 2005). Vitality and learning glean an extended pool of resources, which turns into a better performance (Bruch and Ghoshal, 2003).

When employees thrive at work, by the means of having a pool of resources, they tend to commit to the organization, which makes them perform their jobs in a more attached way (Walumbwa et al., 2018). Moreover, past studies confirmed that when employees thrive at work, their tendency of performing job tasks becomes more efficient and effective (Paterson et al., 2014; Frazier and Tupper, 2018; Walumbwa et al., 2018).

**H3:** *TAW has a positive influence on task performance*.

### The Mediating Role of Thriving

Thriving is a psychological state comprising a joint experience of learning and vitality (Spreitzer et al., 2005). Individuals need psychological resources to experience learning and vitality. Stressors play a negative role in affecting individuals' personal, social, and work-life (Polatc and Özyer, 2015). We postulated that stressors (RC and perceived workload) affect thriving adversely which leads to a declined task performance. Due to the reason that stressors tend to reduce the motivation to learn and better performance (LePine et al., 2004), this perception of negative factors (RC and perceived workload) will lessen the possibility of learning and vitality (Flinchbaugh et al., 2015) and result in lower task performance. Past literature suggested that TAW is a strong mediating mechanism between negative elements and employees' performance.

The COR theory underscores the mechanism of behavioral outcomes at work due to stressful features (workload and RC), which cause in loss of resources (Hobfoll et al., 2018). COR suggests that workplace stressors, i.e., RC and perceived workload have negative effects on task performance by depleting the resource stock (Quinn et al., 2012; Halbesleben et al., 2014). Previous studies have postulated that stressors affect employees' work performance adversely (Tang and Chang, 2010).

Role conflict and work overload tend to result in a loss in the spiral of resources, which affects thriving adversely. Cullen et al. (2018) explored the adverse effect of role on thriving. This means that individuals, with confusion about whom to receive orders from and with the heavy workload, may not feel thrived.

Individuals with no positive sentiments may not gain a pool of resources that play a vital role in making task performance effective (Beal et al., 2005). On the one hand, learning improves the intellectual competencies of individuals which results in better performance (Rose et al., 2009). Shan et al. (2016) stated that fewer learning opportunities cause a decline in individuals' performance. Extant literature has investigated and confirmed the strong association between learning and employees' effective performance at work (Škerlavaj et al., 2007). Frazier and Tupper (2018) also corroborated that thriving employees are in a better position to perform their tasks effectively. Moreover, Walumbwa et al. (2018) stated that thriving promotes commitment in employees which in turn makes employees centered on performing job tasks effectively.

On the other hand, depletion of resources does not only target the human brain's activation but also physical abilities, so they are less likely to perform with energy and may not learn at the workplace (Mullins et al., 2014), in turn, the efficiency required for performing tasks is declined along with information processing. This suggests that people with low thriving will not be able to perform their tasks effectively and efficiently (Frazier and Tupper, 2018). Putting together, employees exposed to workplace stressors will not be experiencing TAW, which consequently will be exhibited with ineffective task performance.

**H4:** *Thriving mediates the relationship between (a) RC, (b) PWL, and task performance*.

### The Moderating Role of Resilience

Along with thriving, we posed that individuals' ability to cope with stressors, such as RC and perceived workload, is contingent on their level of RES (Flinchbaugh et al., 2015). RES is defined as a psychological resource that acts as a buffering mechanism and allows individuals to bounce back and cope with the negative circumstances (Masten, 2001). COR explicates that RES in individuals not only stops the loss of spiral of resources but also helps them to gain a pool of resources to cope with the negative elements in their environment (Hobfoll et al., 2018). Recent literature evidenced the vital role of RES as a moderator for suppressing the negative association between stressors and thriving (Flinchbaugh et al., 2015). RES has been examined in a variety of life segments and tested for being successful or as a failure, i.e., in a workplace setting (Luthans et al., 2005) and also in the academic domain (Cappella and Weinstein, 2001). In a study of nurses, Tusaie and Dyer (2004) stated that since their job is characterized by stress due to their job routine, that is why RES was much needed to deal with adversity.

Role conflict and workload as stress factors tend to affect employees' psychological states negatively which develops negative emotions. Resilient individuals possess the capacity to recover from stressors and to adjust themselves to the changing work requirement (Niitsu et al., 2017). The damage due to negative stressors can be recovered through RES, which protects from destructive behaviors and diseases (Ryff and Singer, 1996). Connor and Davidson (2003) suggested that resilient individuals buffer the negative consequences arising from negative events by strengthening their physical and psychological health. In the presence of stressors, the reactive measures of RES enable individuals to go above the adverse effects of negative factors (Tugade and Fredrickson, 2004).

It is suggested that the tendency of resilient individuals will be high to prepare for hardships to be faced at the workplace, buffering the negative effects on themselves of any such stressful factors by utilizing the spiral of psychological resources (Fredrickson et al., 2008). In this course, we postulated that the presence of RES as a pool of psychological resources buffers the negative effect on individuals' psychological states.

**H5:** *Resilience moderates the relationship between {(a) RC, (b) PWL}, and thriving, such that these negative relationships will be weaker at higher levels of RES (vs. low)*.

## Methods

### Participants and Procedures

The participants are the front-line hotel workers in the Chinese hotel industry. A simple random sampling technique was used for data collection from 3-star and above hotels. Front-line employees are considered to be in a more advantageous position to have information about customers' preferences (Wu and Chen, 2019). The participants of the study varied from but were not limited to front desk services, customer handling, porters, food, and beverages to housekeeping.

A descriptive research design named a cross-sectional survey design was used for this study. As the first step, we seek consent from the hotel organizations to participate in the survey. Furthermore, the participants who agreed to take part in the survey were approached and explained to them the purpose of the survey. The participants were assured about the confidentiality of their information. The survey questionnaire included demographics (i.e., gender, age, span of employment, and education) and the main variables of this study. To avoid the common method variance (Podsakoff et al., 2003), the collection of data was carried out at two points of time and from two sources, i.e., employees and their immediate supervisors. The average number of workers being supervised by each supervisor varied from 8 to 12.

The survey was administered to 400 employees to record their responses about RC, perceived workload, thriving, and RES. In total, 348 questionnaires were received out of which 331 were useful. At time 2, we got the responses from supervisors about employees' task performance, yielding a response rate of 82.75% through matching supervisor-employee questionnaires. This study used a researcher-generated code strategy to match the responses from employee-supervisor dyads. Each participant was given a unique code, and the same code was provided to his/her supervisor for compilation of the responses by employees and supervisors by matching these unique codes.

### Control Variables

We controlled the effects of demographic variables. We coded these variables as gender (1 = male, 2 = female), age (1 = 21–25 years, 2 = 26–30 years, 3 = 31–35 years, 4 = 36–40 years, and 5 = 41 and above), education (1 = middle school, 2 = high school, 3 = college, 4 = bachelors, and 5 = master or higher), and tenure (1 = 1–5 years, 2 = 6–10 years, 3 = 11–15 years, 4 = 16–20 years, and 5 = 21 and above years).

### Measures

This study uses a mature scale and takes great care while designing the survey ensuring the quality of the study. We used the back-translation method (Brislin, 1970) because the original language of the measures was English. A five-point Likert scale ranges from 1 = strongly disagree to 5 = strongly agree. We controlled the effects of the demographic variables due to the chances of their dominance of individuals' behavioral aspects (Ng and Feldman, 2012).

#### RC and PWL

We measured the concept of RC through 8-items and perceived workload through 4-items by Allen et al. (2008). The sample questions for the RC and perceived workload were “I receive an assignment without the manpower to complete it” and “I feel that the number of requests, problems, or complaints I deal with is more than expected” with the coefficient α = 0.907 (RC) and α = 0.812 (perceived workload), respectively.

#### Resilience

We measured the level of RES as employees' coping strategy through 6-items borrowed from Smith et al. (2008). The sample items are “It does not take me long to recover from a stressful event” with the coefficient α = 0.888.

#### Thriving at Work

We measured employees' sense of learning and vitality through 10-items borrowed from Porath et al. (2012). The sample item included “I am looking forward to each new day.” The coefficient α is 0.941.

#### Task Performance

We measured the effectiveness of employees' task performance through supervisor-rated 4-items adapted from Eisenberger et al. (2001). The sample items included “This employee meets formal performance requirements of the job” with the coefficient α = 0.868.

## Results

### Correlation

Table 1 presents the descriptive statistics as mean, standard deviations, and correlations. As expected, RC (*r* = −0.372, *p* < 0.001) and PWL (*r* = −0.361, *p* < 0.001) were negatively related with thriving. TAW was positively related with TP (*r* = 0.439, *p* < 0.001).

**Table 1:** Correlation.

**Variables**	**M**	**SD**	**VIF**	**RC**	**PWL**	**TAW**	**RES**	**TP**
**RC**	3.415	0.897	1.330	**0.746**				
**PWL**	3.182	0.881	1.464	0.437^**^	**0.729**			
**TAW**	3.843	0.921	1.283	−0.372^**^	−0.361^**^	**0.774**		
**RES**	3.020	0.952	1.237	−0.159^**^	−0.396^**^	0.307^**^	**0.780**	
**TP**	3.758	0.839	1.291	−0.439^**^	−0.348^**^	0.439^**^	0.085	**0.783**

** *Correlation is significant at the 0.01 level (2-tailed)*.

*RC, role conflict; PWL, perceived workload; TAW, thriving at work; RES, resilience; TP, task performance; M, mean; SD, standard deviation; VIF, variance inflation factor*.

*The square root of AVE is presented in bold in diagonals*.

### Validity and Reliability

The CFA was performed to assure the validity of constructs. We checked several model fitness indexes for our measurement model in AMOS (Kline and Kline, 1998). A good model fit was observed for 5-factor χ^2^ = 579.832, df = 454, χ^2^/df = 1.277, SRMR = 0.055, TLI = 0.977, CFI = 0.979, and RMSEA = 0.029. The composite reliability (CR) showed the values 0.819–0.937 which are >0.70 (Hair et al., 2011). Moreover, results showed “α” values varying from 0.812 to 0.941, which are >0.70.

The value of average variance extraction varied from 0.532 to 0.613, which is >0.50 (Fornell and Larcker, 1981). The condition of discriminant validity was fulfilled first, the square root of AVE was greater than the correlation coefficient of other variables (Fornell and Larcker, 1981), and second, the correlation coefficients were below 0.85 (Kline, 2005).

### Hypothesis Testing

**Figure 1:**
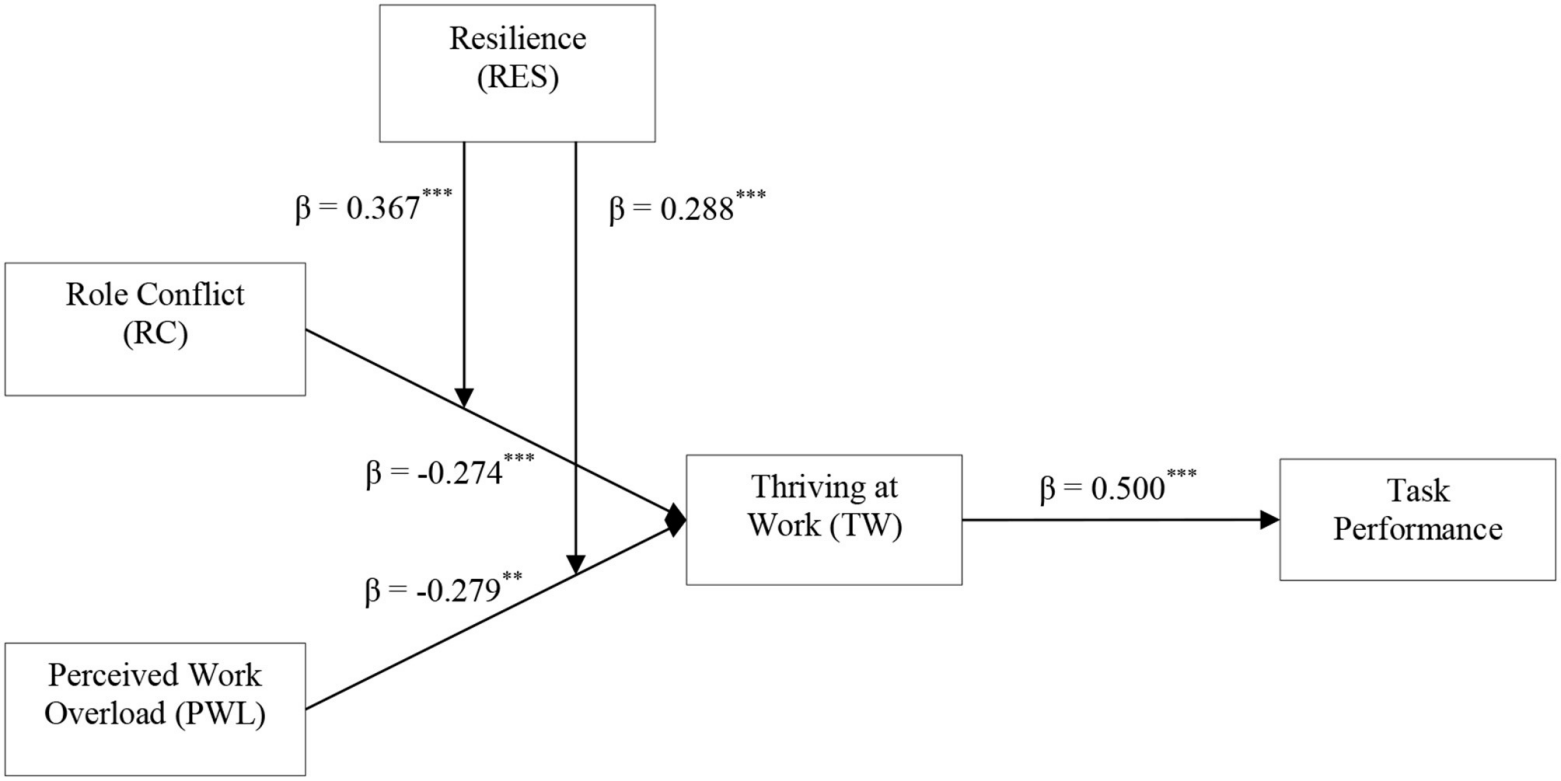
Theoretical framework. **p < 0.01;***p < 0.001.

The hypotheses for this study were tested using structural equation modeling (SEM) due to its ability to examine the multiple variables at the same time. As the first step, we tested the multicollinearity among variables through the value of (VIF = 1.237-1.464). Table 1 which was less than the standard value of 3. Hypotheses 1, 2, and 3 were tested through SEM (AMOS). H1 and H2 proposed that RC and PWL are negatively associated with thriving. H3 proposed that TAW is positively related to task performance. The results provide support for these relationships (β *RC*→*TAW* = −0.274, *p* < 0.001), (β *PWL*→*TAW* = −0.279, *p* < 0.01), and (β *TAW*→*TP* = 0.500, *p* < 0.001) (Figure 1).

H4a and H4b proposed that thriving mediates the link between (a) RC, (b) PWL, and TAW. We employed the bias-corrected method with 5,000 bootstrap samples at a 95% confidence interval (CI) (Hayes, 2013). The results showed support to the proposed hypotheses (β *RC*→*TAW*→*TP*) = −0.137, [CI: −0.211, −0.080], and (β *PWL*→*TAW*→*TP* = −0.140, [CI: −0.237, −0.063]. The CI does not include any zero between BOOT CI [LLCI, ULCI], which evidences the significant indirect effects (Table 2). The result showed a good fit for the mediation model χ^2^ = 402.239, df = 295, χ^2^/df = 1.364, SRMR = 0.081, TLI = 0.976, CFI = 0.978, and RMSEA = 0.033.

**Table 2:** Indirect paths.

**Path**	**Coefficients**	**Boot SE**	**CI. 95%**	**Relationship**
RC—->TAW–>TP	−0.137	0.033	{−0.211, −0.080}	Supported
PWL–>TAW–>TP	−0.140	0.044	{−0.237, −0.063}	Supported

**Figure 2:**
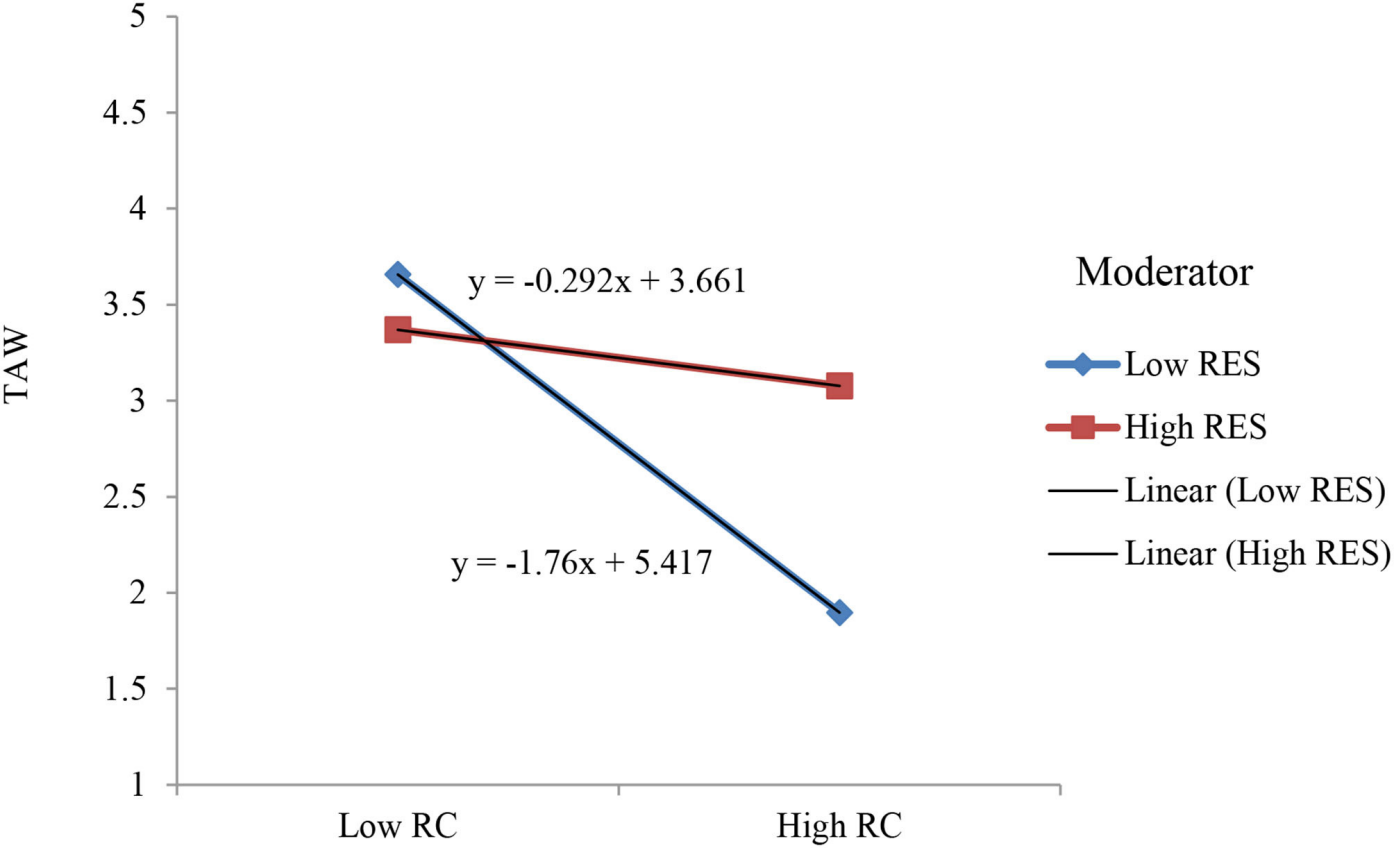
Resilience (RES)'s moderating effect on thriving at work (TAW) through role conflict (RC).

**Figure 3:**
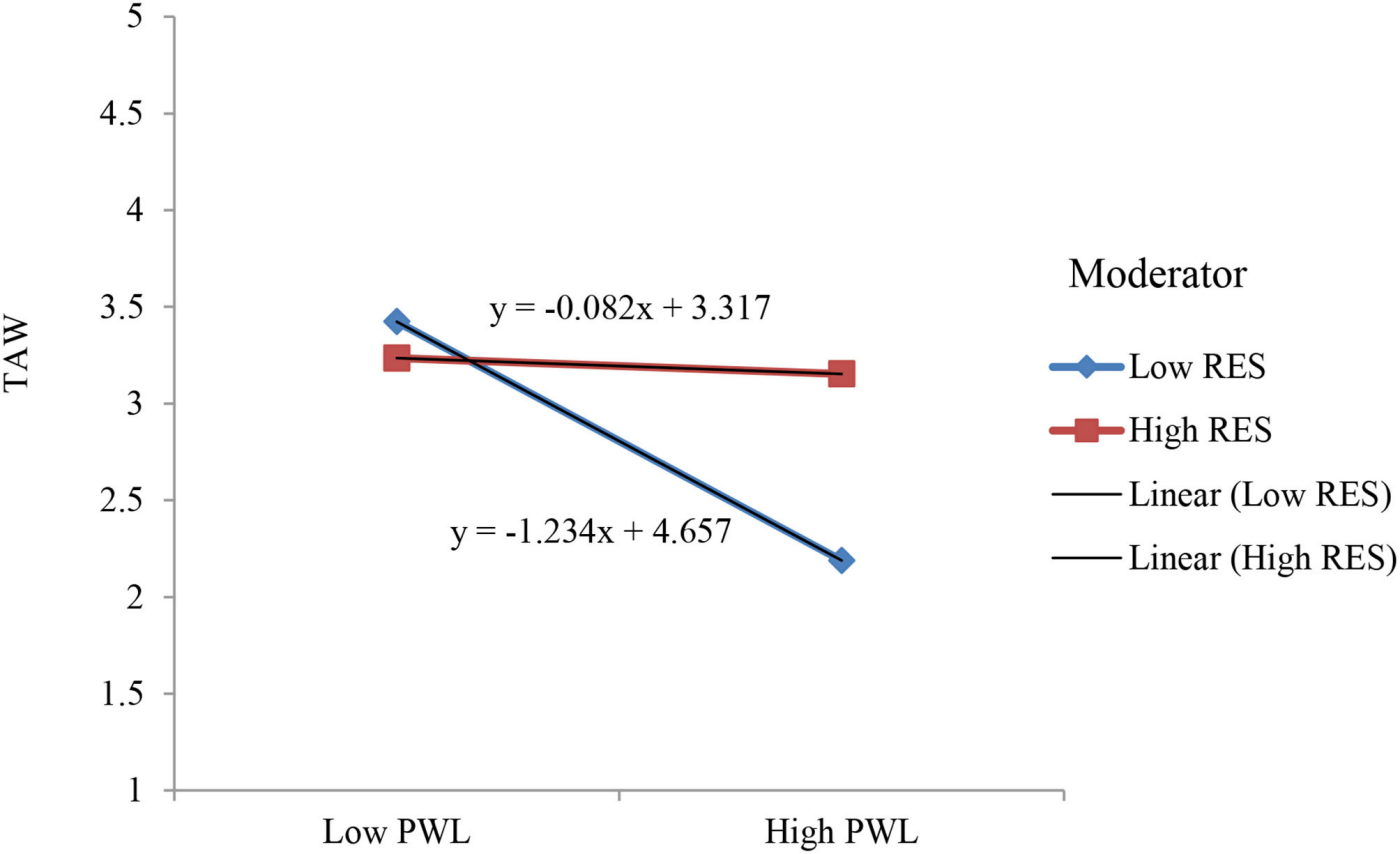
Resilience's moderating effect on TAW through perceived workload (PWL).

To test the moderating effects of RES (H5a and H5b), we used PROCESS macro 3.3 model 1 (H5a and H5b) with 5,000 bootstrap samples at 95% CI (Hayes, 2013). For RC and PWL, we tested the separate moderating effect of RES. Results show that RES significantly moderated the relationships among RC, PWL, and thriving (β *RES* X *RC* → TAW = 0.367, [0.261, 0.474], *p* < 0.001) and (β *RES* X *PWL*→*TAW* = 0.288, [0.177, 0.398], *p* < 0.001), thus supports H5a and H5b (Figure 1). The results show that the negative relationship between {RC and perceived workload} and thriving was significant at low level of RES {β = −0.949, *t* = −9.310, *p* < 0.001}, {β = −0.671, *t* = −7.331, *p* < 0.001} rather than high level of RES {β = −0.0915, *t* = −1.509, ns}, {β = 0.001, *t* = 0.011, ns}, respectively. Figures 2, 3 show the plotting of interaction graphs.

## Discussion

This study discusses in what way the job stressors affect individuals' thriving based on the COR theory, which suggests that individuals need resources for the accomplishment of their tasks, and the absence of which can be resulted in a loss in a spiral of resources. The results showed that RC and perceived workload have a negative relation with TAW. It shows a consistency with previous literature which shows that RC was negatively related to employees' behavioral outcomes, such as work engagement, job satisfaction, and organizational commitment (Maden-Eyiusta, 2019). The perceived workload has also been evidenced to corroborate the findings of previous literature, such as the negative relationship between workload and creative behavior (De Clercq and Belausteguigoitia, 2019).

Results show that thriving is positively associated with individuals' task performance consistent with the findings (Frazier and Tupper, 2018; Elahi et al., 2020). Moreover, results showed that RES has a significant moderating effect on the relationships between RC, perceived workload, and TAW. RES has more strongly moderated the relationship between RC and thriving as compared with perceived workload and TAW. It suggests that individuals' RES recovers them from the stress they face from conflict of roles and perceived workload. Moreover, individuals facing RC are still in a better position to recover from this stress as compared with the workload for which RES works at a slightly low level.

### Theoretical Implications

Based on our results, several theoretical implications can be seen. It is the first study to our knowledge that explores the potential impact of RC and perceived workload on TAW. By employing COR theory, it advocates that individuals need a pool of resources to accomplish their jobs and to gain more resources. RC and perceived workload, in this regard, play the role of stressors, which cause a loss in a spiral of resources which ultimately reduce the individuals' potential to sense learning and vitality.

Second, we examined that thriving enhances the task performance of employees. Thriving employees are nurtured with vitality and learning that helps them to show an enhanced task performance. Third, TAW mediates the link between RC and perceived workload and task performance. It suggests that RC and perceived workload affect the TAW in an adverse way which leads to lower task performance. Fourth, we examined the crucial role of RES as a coping strategy to moderate the negative relationship between RC, PWL, and TAW. Finally, it signifies its contribution by utilizing key implications of COR theory along with the data collected from two sources which helped to avoid common method bias (CMB) (Podsakoff et al., 2003).

### Practical Implications

Along with the theoretical implications, our study offers practical implications too. First, the results showed that RC and perceived workload have a negative association with TAW. It suggests that the hotel managers clarify the roles of each employee and clarify the hierarchy and reporting system and also what type of activities should be assigned and by whom, to avoid this situation. Second, the managers should take care of employees' workload to make them more resourceful so that they not only can manage their tasks but also feel thrived at work. The managers can devise the workload according to the individuals' capacity and compensations. Third, managers should conduct training for all levels so that everybody gets on the same level and to address the gaps arising from the RC and workload so that employees can thrive and exhibit their task performance effectively.

Finally, as previous studies stated that hotel organizations are characterized by heavy workload (Wu and Chen, 2019), and managers should be able enough to provide resources in such a way that every employee should possess a pool of resources so that their learning ability and vitality should be boosted and they can perform well at their jobs.

## Limitations and Future Directions

Even though we employed appropriate theoretical grounding and effective quantitative techniques to strengthen the arguments through results yet, it is not without limitations. First, we could not observe causal inference due to the cross-sectional nature of the data. To counter this problem, a longitudinal study design can be undertaken in the future. Second, we explored our theoretical model in the Chinese hospitality sector, which raises the concern of generalizability due to contextual and cultural differences among eastern and western cultures (Zhang et al., 2019). The scholars can explore this theoretical mechanism in the western context and other sectors to gain cultural and corporate understandings.

Third, even though we collected the dyadic data and two points of time to avoid CMB, yet, it bears a weakness because supervisor-rated task performance may vary from actually performed tasks. We encouraged the scholars to deal with this limitation by evaluating employees' task performance through an interim appraisal program after a detailed discussion with employees about their performance. Fourth, due to the small size of the sample, we cannot generalize the findings at a mass level; however, future studies can mitigate this limitation by working on large data sets. Finally, we have examined how RES can suppress the negative effects of RC and perceived workload on thriving. Future studies can explore the moderating role of RES or other coping strategies between other stressors, such as coronavirus disease (COVID) anxiety and thriving.

## Conclusion

This research was aimed at exploring a framework that can provide useful insights to understand the concept of TAW in connection to stressors. This study has contributed to the existing pool of research from different perspectives: first, by offering different insights about job stressors and TAW; second, by explicating the effects of thriving on task performance; third, by offering useful insights and theoretical support for COR theory; and fourth, by testing the proposed theoretical model in the hospitality industry of China which reveals key insights into the working environment and employees' behaviors.

To start with, the results of this study through the structural equation model showed that factors, such as RC and perceived workload affect TAW, negatively based on COR theory. More closely, we examined the effects of RC and perceived workload and observed that PWL has a slightly higher negative association with TAW which shows that work overload is a greater cause of loss in individuals' resources, which do not let them experience TAW. It suggests that hospitality managers should clarify the role of employees to avoid the collision, and most importantly, lowering the workload of employees will make them in a better position to gain the spiral of resources and to experience TAW.

This study has examined the important relationships between thriving and task performance. We observed that thriving has a positive relationship with task performance. Moreover, the crucial mediating role of thriving bears high importance which links RC, perceived workload, and task performance. The results suggest that RC and perceived workload cause the loss in individuals' resources, which reduces their thriving, leading to lower task performance. This study also entails the crucial moderating role of RES between RC, perceived workload, and TAW. The results showed that resilient individuals are in a better position to cope with the negative consequences of RC and perceived workload on their level of thriving.

## Data Availability Statement

The raw data supporting the conclusions of this article will be made available by the authors, without undue reservation. Requests to access these datasets should be directed to HF, hassaanfarid3@gmail.com.

## Author Contributions

ZH conceived the idea and developed the theoretical framework. HF adopted the methodology and calculated the results. WA and XL completed interoperation and discussions based on statistically analysis. All authors contributed to the article and approved the submitted version.

## Conflict of Interest

The authors declare that the research was conducted in the absence of any commercial or financial relationships that could be construed as a potential conflict of interest.

## Publisher's Note

All claims expressed in this article are solely those of the authors and do not necessarily represent those of their affiliated organizations, or those of the publisher, the editors and the reviewers. Any product that may be evaluated in this article, or claim that may be made by its manufacturer, is not guaranteed or endorsed by the publisher.
